# Antioxidant and antimicrobial activities of *Salsola imbricata* methanolic extract and its phytochemical characterization

**DOI:** 10.1515/biol-2022-1011

**Published:** 2024-12-16

**Authors:** Helmy A. Aamer, Saad F. Elalem, Abdulaziz A. Al-Askar, Omaima A. Sharaf, Mahmoud A. Gaber, Przemysław Kowalczewski, Said Behiry, Ahmed Abdelkhalek

**Affiliations:** Department of Chemistry and Technology of Pesticide, Agriculture Faculty (El-Shatby), Alexandria University, Alexandria, 21545, Egypt; College of Physical Education and Sport Sciences, Al-Mustaqbal University, Babylon, Iraq; Department of Botany and Microbiology, College of Science, King Saud University, P.O. Box 2455, Riyadh, 11451, Saudi Arabia; Department of Agricultural Microbiology, National Research Centre, Cairo, 12622, Egypt; Department of Plant Pathology, Faculty of Agriculture (El-Shatby), Alexandria University, Alexandria, 21545, Egypt; Department of Food Technology of Plant Origin, Poznań University of Life Sciences, Poznań, Poland; Agricultural Botany Department, Faculty of Agriculture (Saba Basha), Alexandria University, Alexandria, 21531, Egypt; Plant Protection and Biomolecular Diagnosis Department, Arid Lands Cultivation Research Institute, City of Scientific Research and Technological Applications, Alexandria, 21934, Egypt

**Keywords:** *Salsola imbricata*, soil-born fungi, HPLC, GC–MS, antioxidant, antimicrobial activity

## Abstract

Methanolic extract from *Salsola imbricata* was investigated for its phytochemical content, antioxidant, and antimicrobial properties against phytopathogenic fungi and bacteria. Phytochemical analysis revealed the presence of saponin, tannins, and alkaloids with 1.25%, 18.8 mg catechin/g of extract, and 9.12%, respectively. Total flavonoid content was 20.8 mg quercetin equivalent/g while total phenolic content was 202 mg gallic acid equivalent/g. Antioxidant activity using the 2,2-diphenyl-1-picrylhydrazyl assay resulted in an IC_50_ value of 48.61 µg/mL, while the phosphomolybdenum method yielded a value of 215.43 mg ascorbic acid equivalent/g of extract. The highest phenolic acids detected in the extract were gallic acid (712.97 µg/g), syringic acid (742.7 µg/g), and caffeic acid (474.70 µg/g) according to high-performance liquid chromatography analysis. Palmitic acid (28.38%) dominated the fatty acids identified by gas chromatography–mass spectrometry, while stigmasterol (8.34%) was the most abundant steroid. At a concentration of 3 mg/mL, the extract showed strong antibacterial activity against *Pectobacterium carotovorum* (10.50 mm), *Ralstonia solanacearum* (9.93 mm), and *Pectobacterium atrosepticum* (8.37 mm). Additionally, the extract significantly suppressed fungal growth of *Rhizoctonia solani* (38.22%) and *Fusarium oxysporum* (33.56%) but showed lower activity toward *Botrytis cinerea* (13.33%) at 5 mg/mL. In conclusion, *S. imbricata* extract exhibited promising antioxidant and antimicrobial properties, making it a potential candidate for further exploration in agricultural applications.

## Graphical abstract

**Figure:**
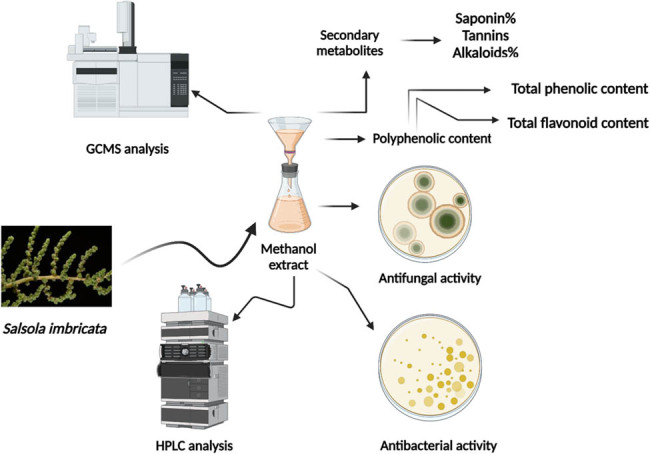

## 1 Introduction

Naturally occurring plant-based chemicals can function as pesticides and mitigate fungal diseases in agricultural settings. Extensive attempts have been undertaken to examine plants to discover alternative natural fungicides that can substitute for the existing synthetic ones, which are linked to issues such as harmful effects on plants, resistance, and revival of pests, toxicity to vertebrates, vast environmental risks, and expensive expenses [1]. Halophytes have a large distribution in Peninsular Arabia and the Mediterranean region. They are utilized in traditional herbal treatments and contribute to the diverse plant resources in these areas. These plant species have a higher prevalence in saline settings and possess exceptional adaptability, enabling them to thrive in typical climatic zones [2,3]. Medicinal plants have been a vital source of therapeutic products for thousands of years, with many contemporary drugs derived from plant sources. Traditionally, these plants are valued for their anti-inflammatory, antioxidant, and antibacterial properties. However, the process of identifying and integrating new compounds into modern therapeutic protocols is complex, time-consuming, and costly. As such, there is a growing focus on studying plant extracts, as their synergistic effects often enhance their therapeutic activity beyond that of individual phytochemicals [4]. *Salsola imbricata*, which is commonly included in camel feed, is not only the most widely used remedy for diarrhea but also has medicinal properties that make it effective in treating dysentery, stomachaches, and worm infestations [5].

The genus *Salsola* plays a crucial role in the plant kingdom because of its wide range of traditional, industrial, and environmental uses [6]. *Salsola* species are widely distributed across temperate zones and constitute approximately 45% of the plant species present in desert environments [6]. They serve as an abundant reservoir of many phytochemical categories, including alkaloids, coumarins, cardenolides, flavonoids, phenolic acids, isoflavonoids, and triterpenoids [7]. A variety of phytochemicals derived from plants, including saponins, flavonoids, and terpenoids, possess advantageous properties that promote human health and can be incorporated into functional foods and supplements [8]. *Salsola* species have a long history of medicinal usage due to their anti-inflammatory, blood pressure-lowering, and immune-boosting properties [9]. *Salsola* species has garnered significant attention from researchers due to its numerous pharmacological effects [10]. Recent research has highlighted the potential of medicinal plants to yield bioactive compounds with antimicrobial and antioxidant properties. For example, Ndezo Bisso et al. [11] demonstrated the strong antioxidant and antimicrobial activities of seven selected medicinal plants, including *Psychotria peduncularis* and *Tristemma mauritianum*, which were rich in phenols and flavonoids. Their study underscores the value of medicinal plants in developing natural alternatives to synthetic chemicals for agricultural and pharmaceutical applications. Numerous antioxidants that function as potent oxygen scavengers can be found in medicinal plants. To avoid the disadvantages of synthetic antioxidants, attention has recently been drawn to antioxidants derived from natural sources [12].

Over the last few years, growing attention has been to developing additional environmentally sustainable phytopathogen control in the global agriculture sector [13]. Incorporating phytochemicals as bio-origin agents, in their raw or refined form, is one of the possible strategies showing similar exciting outcomes and is getting a lot of interest [14,15,16]. Phytochemicals are eco-friendly substances that have residual impacts on the environment, including biodegradability, phytotoxicity, and a lack of long-lasting impact [17]. Notwithstanding, a lot of the active substances in these natural extracts are essential for bioprotection [18]. Most of the diverse array of small molecules, or secondary metabolites, made by plants act as safeguards against different microbial risks, pests, and herbivores [19]. Among the secondary metabolites produced in the plant world, phenolic acids are known for their abundant presence [20]. Interestingly, phytochemicals that plant “defense systems” against fungal diseases are primarily based on their diversity [19]. Phenolic compounds can break down the structure of these pathogens directly by changing their morphology, ultrastructure, and physiological processes [16,18]. They can also do this indirectly by making plants resistant to them throughout their systems [21].

Chemical fungicide resistance is a growing concern due to the significant harm caused by plant-pathogenic bacteria and fungi to crops worldwide [22]. These diseases include soft rot, bacterial wilt, vascular wilt, damping-off, root rot, and stem canker diseases [23]. The widespread use of chemical fungicides has raised environmental and health concerns due to their long-lasting nature [24,25]. To address these issues, it is crucial to explore natural sources, like plant extracts, for antimicrobial agents that can counteract these diseases while mitigating environmental impacts and addressing resistance concerns. Research on bioactive substances derived from plant extracts has promising prospects for developing sustainable and ecologically sound substitutes for chemical fungicides, protecting agricultural systems, and promoting human well-being. The current study aims to investigate the phytochemical composition of the halophyte *S. imbricata*, with a specific focus on important components including phenolic content, flavonoid content, and other significant secondary metabolites. In addition, our objective is to do gas chromatography–mass spectrometry (GC–MS) analysis on the aqueous methanolic extract to determine the chemical components that are present. Furthermore, we are investigating the extract’s biological properties, particularly its efficacy against specific phytopathogenic bacteria and fungi, as well as its antioxidant capabilities. By conducting this comprehensive analysis, our study seeks to address the gap in research on *S. imbricata* as a potential source of natural pesticides and its broader medicinal and agricultural applications.

## 2 Materials and methods

### 2.1 Plant material and extraction process

The *S. imbricata* plants were collected at the flowering stage from the El-Hamam region of Matrouh Governorate, Egypt, at coordinates (30°50′22.8″N 29°23′55.9″E) and then grabbed to the Department of Plant Production for plant specimen identification and deposited in the respiratory herbarium of the Faculty of Agriculture (Saba Basha), Alexandria University, Alexandria, Egypt, under voucher number 7706. The plant specimens’ aerial parts were meticulously cleansed using tap water to remove any dust or debris adhering to their surface. After air-drying at ambient temperature in a shaded area for 2 weeks, the desiccated substance was pulverized into a fine powder using a mill, yielding particles with a size of 80 mesh. One kilogram of this fine powder was steeped in 3 L of 80% methanol for 48 h, with occasional agitation. Following the maceration procedure, the extract was filtered using the filter paper of Whatman No. 1 under vacuum conditions, the filtrates were consolidated and evaporated using a rotary evaporator. The dark brown, shapeless powder obtained from the aqueous methanol extract was kept at 4°C [26,27].

### 2.2 Phytochemicals quantification

#### 2.2.1 Total phenolic content (TPC)

The Folin–Ciocalteau reagent was used to quantify the TPC of the extract [28]. Gallic acid was chosen as the standard reference, and the plant extract was dissolved in methanol. The Folin–Ciocalteau reagent was diluted tenfold using deionized water. The reaction mixture, consisting of 0.5 mL of the extract solution and 2 mL of the diluted Folin–Ciocalteau reagent, was incubated for 3 min at 26 ± 2°C. Subsequently, a 1 mL solution of sodium carbonate 7.5% (w/v) was added, and the solution underwent incubation at 40°C for 30 min. The samples were measured for absorbance at a wavelength of 765 nm. A blank solution of deionized water was used as a standard instead of the plant extract. To find the TPC values, a calibration curve made with gallic acid was used as a guide. Data were presented in mg of gallic acid equivalents per gram of extract.

#### 2.2.2 Flavonoid content

With a few minor modifications, the aluminum chloride method – which was described by Woisky and Salatino [29], was used to assess the total flavonoid concentration in the plant extract. Quercetin was used as the standard reference. The process began with dissolving 1 mL of the extract in 2 mL of a 2% methanol-based aluminum chloride solution. The solution was vigorously shaken and then kept at 25 ± 2°C for 1 h in the absence of light. Afterward, the amount of light absorbed by the combination was determined at a wavelength of 420 nm. The flavonoid concentration was quantified by comparing it to a quercetin calibration curve. Data were presented in milligrams of (mg QE/g) of quercetin equivalents per gram of extract. The stated findings correspond to the mean value derived from three separate measurements conducted independently.

#### 2.2.3 Tannin content

The vanillin test created by Ksouri et al. [30] was used to measure the total amount of condensed tannins in an aqueous methanolic extract of *S. imbricata*. By using the reaction between tannins and vanillin in an acidic setting, this method creates a pink-red complex. To prepare triplicate samples, combine 200 µL of extract solution in methanol with 3 mL of 4% vanillin solution in methanol and 1.5 mL of HCl. After 15 min at room temperature, each mixture’s absorbance was measured against a methanol blank at 500 nm. (+)-Catechin was used as the standard, so total condensed tannins could be calculated as mg (+)-catechin equivalent per gram of extract.

#### 2.2.4 Total saponin

For the investigation of total saponins, we followed the method of Obadoni and Ochuko [31] with minor modifications. One gram of dry extract was initially suspended in distilled water, and defatting was carried out twice with diethyl ether (20 mL each) in a separating funnel, followed by discarding the ether layer. Subsequently, the remaining aqueous layer underwent extraction with *n*-butanol. A 10 µL of 5% NaCl was used to wash the *n*-butanol extracts twice. The resulting solution was concentrated to dryness. The saponin content was then determined using the following formula:

\[ \% \text{Saponin content}\hspace{.25em}=\hspace{.25em}{[}\text{Final weight of the sample}/\text{Initial weight of extracts}]\hspace{.25em}\times \hspace{.25em}100]\]

#### 2.2.5 Alkaloids content

The alkaloid content was measured using the Richardson and Harborne [32] technique. In a sealed 250-mL beaker, 1 g of dried Salsola methanolic extract was combined with 100 mL of 10% acetic acid (dissolved in ethanol). The mixture was left to incubate for 4 h. The extract was further filtered and condensed, and then, ammonium hydroxide was added progressively until precipitation occurred. Subsequently, the precipitate was filtered with Whatman filter paper and purified with diluted ammonium hydroxide. The remaining substance was measured in weight and subjected to dehydration at 40°C. The alkaloid content was calculated using the following formula:

\[ \% \text{Alkaloid}\hspace{.25em}=\hspace{.25em}{[}\text{Final weight of the sample}/\text{Initial weight of the extract}]\hspace{.25em}\times \hspace{.25em}100]\]

### 2.3 HPLC analysis of polyphenolic compounds

The phenolic content of the extract was analyzed using an Agilent 1260 Infinity high-performance liquid chromatography (HPLC) Series, which consisted of a quaternary pump and a Zorbax Eclipse Plus C18 column (100 mm × 4.6 mm i.d.). A gradient elution system with three mobile phases was used for the separation process. These were (A) HPLC-grade water with 0.2% H_3_PO_4_ (v/v) in it, (B) methanol, and (C) acetonitrile. The column temperature was maintained at 30°C, and the flow rate was kept constant at 1 mL/min. A 25 µL of sample extract was injected, and compound detection was achieved using a UV detector set at 284 nm. To detect the specific chemical, stock solutions were diluted with methanol until the final concentration was 50 μg/mL. This was then used to make real reference samples. The standard references, including gallic acid, chlorogenic acid, catechin, methyl gallate, caffeic acid, syringic acid, rutin, ellagic acid, coumaric acid, vanillin, ferulic acid, naringenin, daidzein, quercetin, and cinnamic acid, purchased from Sigma-Aldrich (Germany), were compared with the retention times of the analytes obtained from the sample extract. Finally, quantification was determined based on peak area computation using ClarityChrom^®^ Version 7.2.0 software.

### 2.4 GC–MS analysis

The extract phytochemical composition was analyzed using the Thermo Scientific Trace GC Ultra ISQ mass spectrometer. The analytical approach utilized a TraceGOLD TG-5MS direct capillary column (30 m × 0.25 mm × 0.25 μm film thickness). The methanolic extract was dissolved in analytical-grade methanol. Starting at 50°C, the temperature of the column oven was raised at a rate of 5°C/min until it reached 230°C, and then, it was maintained at that level for 2 min. Then, the temperature went up to 290°C and kept it for 2 min. The injector transfer line temperature was maintained at 250°C, while the mass spectrometer transfer line temperature was maintained at 260°C. At a temperature of 250°C, a 1/4 volume of helium was injected with a split ratio of 1:30 as the carrier gas. Under electron ionization mode, the mass spectrometer was operated at a temperature of 200°C and an energy level of 70 eV. A scan range of 40–1,000 m/z was set up for the mass spectra analysis. To determine the compounds, we used mass spectra and retention time comparisons with information from the Wiley and NIST MS library databases.

### 2.5 Antimicrobial activities assay

#### 2.5.1 Antifungal assessment

*Fusarium oxysporum* (Acc# OQ820156), *Rhizoctonia solani* (Acc# OQ880458), and *Botrytis cinerea* (Acc# OR116485) are three common phytopathogenic fungi that were previously identified and used to test the extract’s antifungal activity [33,34]. In addition to the extract, copper hydroxide, a common agricultural fungicide, was tested as a positive control at a concentration of 250 µg/mL. To test the effect of plant extract against the mycelial radial growth, the extract was dissolved in DMSO, then mixed with sterile PDA media, and poured into 9-cm Petri plates to achieve different concentrations ranging from 1 to 5 mg/mL. For each fungus, agar plugs with actively growing fungal hyphae (5 mm in diameter) were carefully put in the center of Petri plates. The diameter of colony growth was measured after incubation at 27°C without light until fungal growth in the negative controls covered the whole surface of the Petri plate. The proportion of mycelial growth inhibition was calculated using the following formula:

\[\text{Mycelial growth inhibition}( \% )\hspace{.25em}={[}(\text{DC}-\text{DT})/\text{DC}]\hspace{.25em}\times \hspace{.25em}100,]\]

where DC represents the average diameter of fungal growth observed in the control group, whereas DT denotes the mean diameter measured in the test extract treatment. All experiments were triplicated.

#### 2.5.2 Antibacterial assessment

In the investigation of antimicrobial activity using the paper disc diffusion technique, the methanolic extract underwent sterilization through filtration with a 0.22 µm Millipore filter. Concentrations of 0.5, 1, 2, and 3 mg/mL were applied by transferring 20 µL of the respective extract concentrations onto pre-sterilized 5 mm filter paper discs. The bacterial isolates *P. carotovorum* (Acc# MN598002), *P. atrosepticum* (Acc# MG706146), and *R. solanacearum* (Acc# LN681200) isolates were previously identified [35]. Using sterile cotton swabs, the bacterial cultures were equally dispersed on nutrient agar plates. The discs, along with positive controls containing streptomycin (10 µg/disc) and negative controls with DMSO (20 µL/disc), were then placed on the agar surface. The diameters of the inhibition zones surrounding each disc were measured in mm after incubation at 37°C for 24 h. To be sure the results could be repeated, we ran each treatment three times.

### 2.6 Antioxidant activity (DPPH)

The electron-donating capacity of the extract was evaluated using the 2,2-diphenyl-1-picrylhydrazyl (DPPH) radical scavenging assay, as outlined in the procedure by Bakar et al. [36]. The extract was dissolved in methanol at varying concentrations (2.4–175 µg/mL), and a 40 µg/mL DPPH solution was prepared in methanol. In triplicate, 200 µL of each extract concentration or ascorbic acid (positive control) was mixed with 1.8 mL of the DPPH solution. The solutions were agitated and kept in a dark environment for 30 min at 25°C. The optical density at a wavelength of 517 nm was determined using 1 cm cuvettes, comparing the sample to a blank solution without DPPH. The percentage of free radical scavenging activity was determined using the formula: [control absorbance – sample absorbance]/[control absorbance] × 100. The control was established using methanol instead of the extract solution. The activity vs concentration was plotted, and the IC_50_ (concentration inhibiting 50% of radicals) was determined.

### 2.7 Total antioxidant capacity (TAC)

The phosphomolybdenum assay was employed to test the TAC, which takes into account how well it can change molybdenum (VI) into molybdenum (V). This reduction, facilitated by antioxidants in the sample, leads to the formation of a green phosphate/Mo(V) complex, whose intensity is directly proportional to the TAC. Following the method outlined by Prieto et al. [37], the phosphomolybdate reagent, consisting of 28 mM sodium phosphate, 0.6 M sulfuric acid, and 4 mM ammonium molybdate, played a pivotal role in this assay. The extract, dissolved in methanol, underwent testing, while ascorbic acid served as a standard (20–200 µg/mL). The reaction mixtures consisted of 400 µL of either the extract solution or ascorbic acid solution, together with 3.1 mL of phosphomolybdate reagent. These mixtures were vigorously shaken and then incubated at 95°C for 90 min while being kept in the dark. Once the solutions were cooled, the absorbance of each solution that had been incubated was measured at a wavelength of 695 nm, with a blank serving as a reference. The extract’s overall antioxidant capability was quantified as an ascorbic acid equivalent (AAE) per gram of extract, providing a comprehensive measure of its antioxidant potential.

### 2.8 Statistical analysis

The data analysis for the antimicrobial activity of *S. imbricata* extract against fungal and bacterial strains was conducted using SAS 9.2 in a completely randomized design, employing a one-way analysis of variance. Mean comparisons were performed using Tukey’s test at a significance level of *P* < 0.05. The standard deviation (±SD) was calculated for both the antimicrobial activity studies and the quantification of saponins, tannins, alkaloids, total phenolics, and total flavonoids. All experiments were carried out in triplicate.

## 3 Results

### 3.1 Estimate the quantity of saponin, tannins, alkaloids, total phenolic, and total flavonoid

The aerial part of *S. imbricata* was macerated and extracted with 80% methanol as a solvent. This gave a yield of 8.37 g/100 g of dry matter. A quantitative analysis was done on the extract to look at important secondary metabolites from different groups of phytochemicals. The comprehensive data, summarized in Table 1, present the phytochemical constituents in dry extract. There were large amounts of secondary metabolites in the extract. The saponin content measured value was 1.25%, while the alkaloid content estimated value was 9.12%. Tannins were also present, with a concentration of 18.8 mg catechin/g extract. Furthermore, the TPC was determined to be 202 mg GAE/g extract, while the total flavonoid content (TFC) was 20.8 mg QE/g extract. These findings contribute valuable insights into the chemical composition of the *S. imbricata* extract, emphasizing its potential significance in various biological and pharmacological applications.

**Table 1:** Bioactive components profiles of *S. imbricata* methanolic extract

Class	Estimated parameter	Concentration ± SD*
Secondary metabolites	Saponin%	1.25 ± 0.05
	Tannins	18.8 ± 0.09
	Alkaloids%	9.12 ± 0.15
Polyphenolic content	Total phenolic content (mg GAE/g)	202 ± 2.65
	Total flavonoid content (mg QE/g)	20.8 ± 0.21

*SD, standard deviation; saponin and alkaloids expressed as percentage %; tannins expressed as mg catechin/g of extract); GAE expressed as gallic acid equivalent; QE expressed as quercetin equivalent.

### 3.2 Antioxidant activity evaluation

The antioxidant capability of the extract was assessed using two different methods. Through phosphomolybdenum-based assay, the extract has a total antioxidant capacity of 215.43 mg AAE/g of extract. The second method was the radical scavenging activity using the DPPH method, which uses ascorbic acid as a reference. The IC_50_ of the extract was 48.61 ± 0.46 µg/mL compared to ascorbic acid which was 7.81 ± 0.25 µg/mL.

### 3.3 HPLC analysis of phenolic and flavonoids

**Figure 1:**
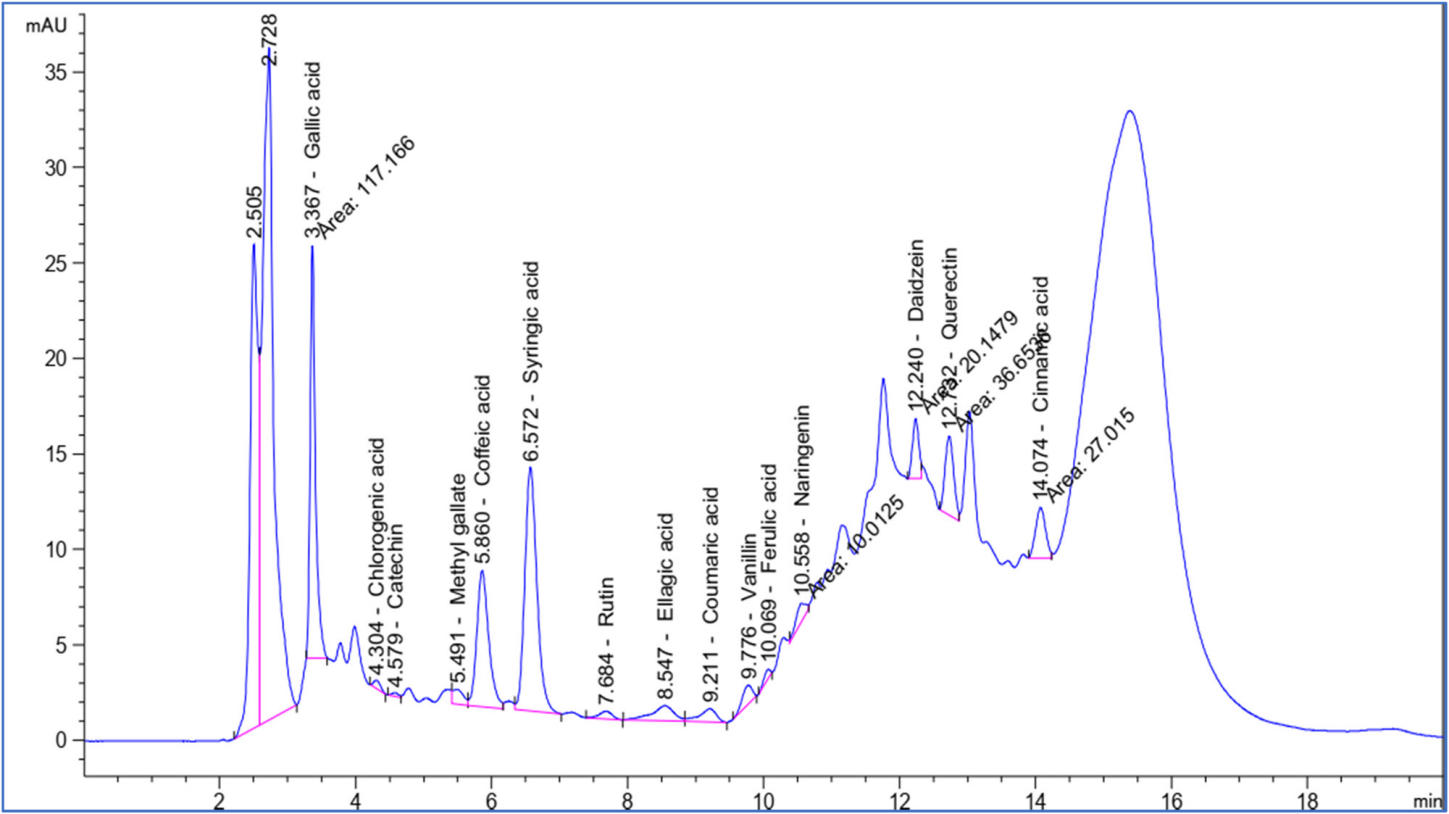
HPLC chromatogram of polyphenolic compounds analysis of *S. imbricata* methanolic extract.

The polyphenolic profile identified in the methanolic extract of *S. imbricata* using HPLC is presented in Figure 1 and Table 2. The highest concentration was observed for syringic acid at 742.71 µg/g, followed closely by gallic acid with a concentration of 712.97 µg/g. Caffeic acid was also present at a substantial concentration of 474.70 µg/g, while quercetin showed a concentration of 331.69 µg/g. Ellagic acid and naringenin were detected at 228.91 and 72.80 µg/g, respectively. Moderate concentrations were found for rutin (41.20 µg/g), methyl gallate (31.16 µg/g), and vanillin (29.65 µg/g). Compounds such as chlorogenic acid, catechin, coumaric acid, daidzein, and cinnamic acid were present in lower concentrations, ranging from 14.18 to 33.79 µg/g. This highlights the significance of high-performance liquid chromatography in precisely defining and measuring these bioactive components in plant extracts.

**Table 2:** The polyphenolic profile identified in the methanolic extract of *S. imbricata* using HPLC

No.	Compounds	RT (min)	Concentration (µg/g)
1	Gallic acid	3.367	712.97
2	Chlorogenic acid	4.304	27.86
3	Catechin	4.579	21.70
4	Methyl gallate	5.491	31.16
5	Caffeic acid	5.86	474.70
6	Syringic acid	6.572	742.71
7	Rutin	7.684	41.20
8	Ellagic acid	8.547	228.91
9	Coumaric acid	9.211	25.28
10	Vanillin	9.776	29.65
11	Ferulic acid	10.069	14.18
12	Naringenin	10.558	72.80
13	Daidzein	12.24	82.49
14	Quercetin	12.732	331.69
15	Cinnamic acid	14.074	33.79

### 3.4 GC–MS analysis

**Figure 2:**
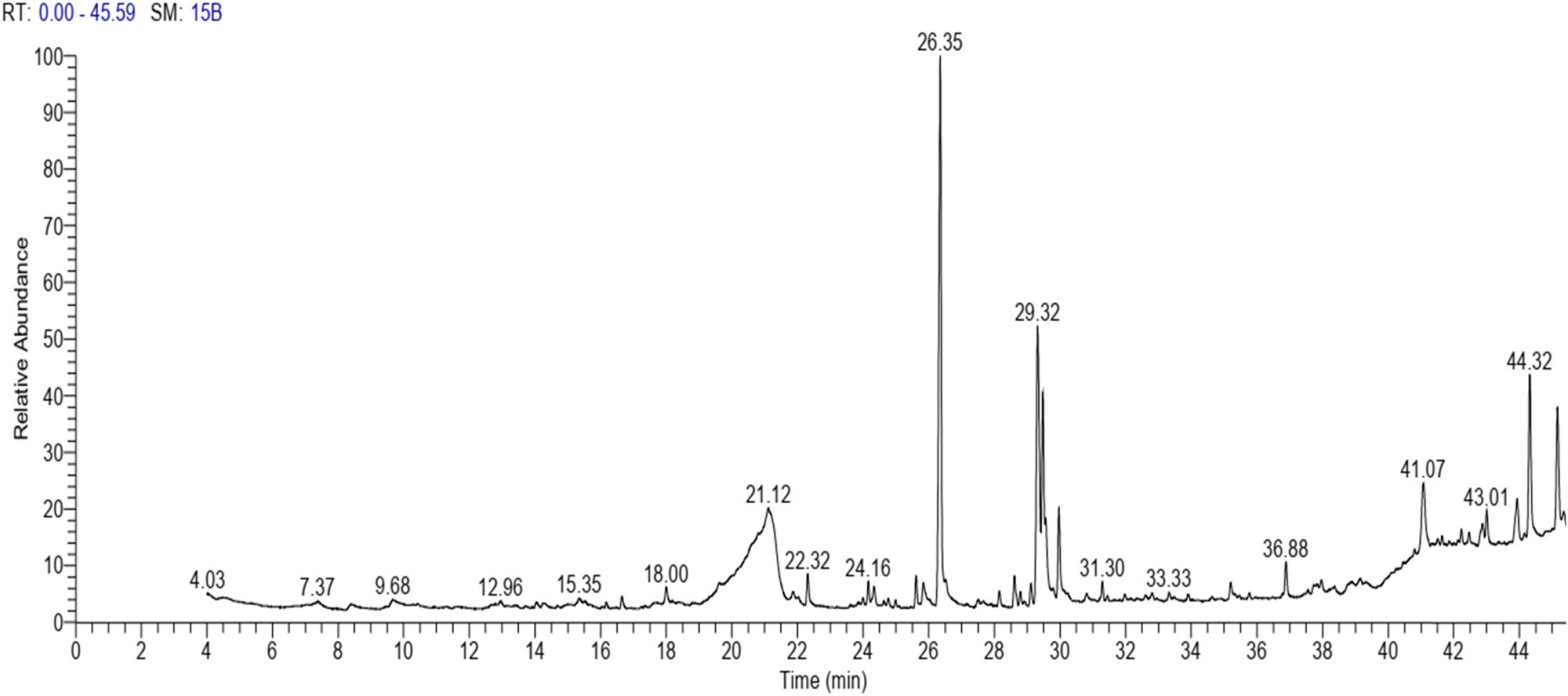
GC–MS chromatogram of *S. imbricata* methanolic extract.

The GC–MS revealed the presence of 19 identified chemicals from different chemical classes. The primary category consisted of fatty acids and fatty acid methyl esters, which accounted for 64.51% of the overall makeup. Palmitic acid was the predominant constituent, accounting for 28.38% of the total composition, with linoleic acid (15.68%), oleic acid (9.53%), and stearic acid (4.16%) following in decreasing order. The composition analysis revealed that steroids constituted 14.92% of the total composition. Among the steroids, stigmasterol was the most abundant, accounting for 8.34%, followed by β-sitosterol at 6.58%. Furthermore, the compound known as triterpenoid methyl ursolate accounted for 6.96% of the total makeup. Figure 2 depicts the GC chromatogram, which visually displays the peaks of the compounds and their elution periods. Table 3 presents the identified substances, including their retention periods, concentrations, and structures. This thorough study, together with the chromatogram, provides insight into the many chemical components found in *S. imbricata*.

**Table 3:** Phytochemical constituents identified in the methanolic extract of *S. imbricata* using GC–MS

RT	Area%	Compounds	Class	Chemical structure
21.19	6.34	d-Glucopyranose,3-*O*-methyl	Methylated carbohydrate	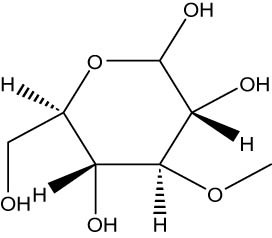
22.31	1.47	Myristic acid	Fatty acid	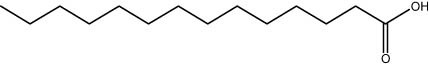
24.16	0.94	2-[(*Z*)-9-Octadecenyloxy] ethanol	Alkene	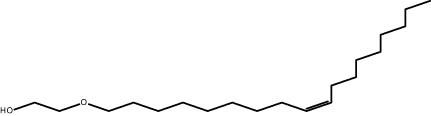
24.34	1.05	Pentadecanoic acid	Fatty acid	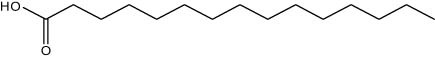
25.61	1.45	Methyl palmitate	Fatty acid methyl ester	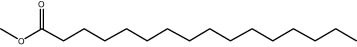
25.83	1.13	Palmitoleic acid	Fatty acid	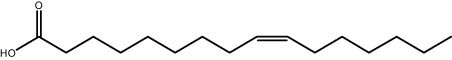
26.35	28.38	Palmitic acid	Fatty acid	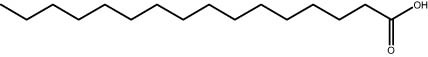
28.61	1.66	Linoleic acid methyl ester	Fatty acid methyl ester	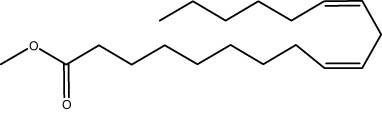
29.11	1.05	Phytol	Terpenoid	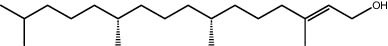
29.31	15.68	Linoleic acid	Fatty acid	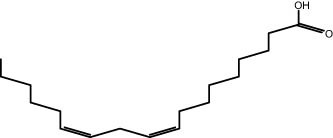
29.56	9.53	Oleic acid	Fatty acid	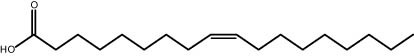
29.96	4.16	Stearic acid	Fatty acid	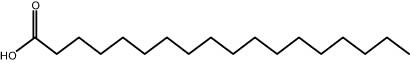
31.3	0.93	Tributyl citrate acetate	Ester	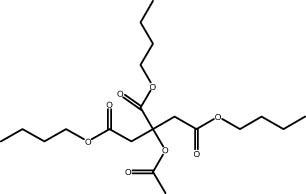
36.88	1.74	Phorbol	Diterpenoids	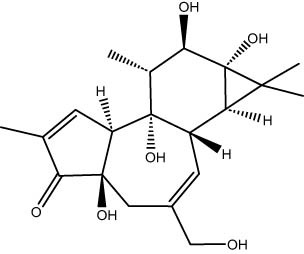
41.06	6.96	Methyl ursolate	Triterpenoid	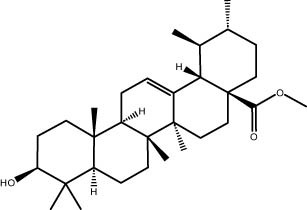
42.88	1.22	Dotriacontane	Alkane	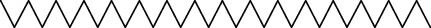
43.01	1.38	Betulin	Triterpene	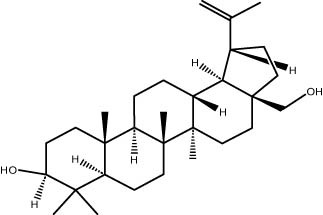
44.32	8.34	Stigmasterol	Steroid	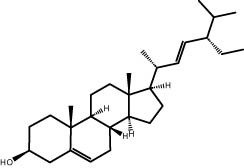
45.16	6.58	β-Sitosterol	Steroid	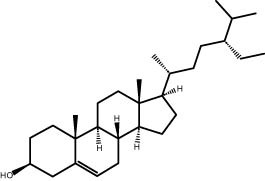

### 3.5 Antimicrobial activity

The extract exhibits significant inhibitory effects on Gram-negative plant pathogenic bacteria, indicating promising antibacterial action (Table 4). The extract inhibited three common pathogens: *P. atrosepticum, P. carotovorum*, and *R. solanacearum*. The antibacterial test exhibited a dose-dependent response, where higher concentrations of the extract led to larger areas of inhibition. The extract worked best against *P. carotovorum*, as evidenced by the inhibition zone’s largest diameter of 10.50 mm at a concentration of 3 mg/mL. *R. solanacearum* exhibited notable susceptibility, as evidenced by an inhibition zone diameter of 9.93 mm at a concentration of 3 mg/mL. *P. atrosepticum* demonstrated comparatively reduced sensitivity, with an inhibition zone diameter of 8.37 mm at the highest concentration.

**Table 4:** Inhibition zone diameter (mm) measurements of phytopathogenic bacteria by methanolic extract of *S. imbricata*

Concentration (mg/mL)	Inhibition zone diameter (mm)*		
	*P. carotovorum*	*P. atrosepticum*	*R. solanacearum*
0.5	7.30 ± 0.04d	0.00 ± 0.00e	0.00 ± 0.00d
1	8.02 ± 0.08c	7.00 ± 0.14d	7.40 ± 0.08c
2	8.98 ± 0.08b	7.75 ± 0.04b	8.79 ± 0.09b
3	10.50 ± 0.11a	8.37 ± 0.06a	9.93 ± 0.15a
Streptomycin (10 µg/disc)	8.28 ± 0.08c	7.37 ± 0.09c	9.73 ± 0.18a

*The means in each column that share the same letter exhibit no significant differences at a probability level of 0.05.

The *S. imbricata* extract was tested for its ability to inhibit the three fungi: *F. oxysporum*, *R. solani*, and *B. cinerea*. The results, shown in Table 5, show that as the concentration of the extract goes up, so does the percentage of inhibited mycelial growth. *R. solani* demonstrated the highest sensitivity to the extract, with a significant inhibition percentage of 38.22% at a concentration of 5 mg/mL. Following closely, *F. oxysporum* displayed notable sensitivity, with an inhibition percentage of 33.56% at the same concentration. In contrast, *B. cinerea* demonstrated lower sensitivity to the extract, exhibiting only a 13. 33% inhibition percentage at the highest concentration. Surprisingly, the extract did not seem to have any effect on *B. cinerea* at concentrations of 1, 2, and 3 mg/mL.

**Table 5:** *In vitro* growth inhibition of phytopathogenic fungi by methanolic extract of *S. imbricata*

Concentration (mg/mL)	Inhibition percentage (%)*		
	*F. oxysporum*	*R. solani*	*B. cinerea*
1	14.89 ± 3.57e	16.22 ± 3.20d	00.0 ± 0.00d
2	16.22 ± 3.39e	20.89 ± 3.18d	00.0 ± 0.00d
3	22.67 ± 1.69d	26.44 ± 2.65c	00.0 ± 0.00d
4	28.67 ± 1.83c	33.78 ± 3.00b	9.56 ± 3.39c
5	33.56 ± 1.99b	38.22 ± 2.68b	13.33 ± 2.48b
Copper hydroxide (250 µg/mL)	66.67 ± 1.67a	78.00 ± 1.65a	61.11 ± 1.76a

*The means in each column that share the same letter exhibit no significant differences at a probability level of 0.05.

## 4 Discussion

*Salsola* is a notable genus of halophytic plants that fall within the Amaranthaceae family. These plants are acknowledged for containing compounds that have antioxidant characteristics and exhibit biological activity [6]. Multiple investigations have already been carried out regarding the fungicidal properties of botanical extracts [8]. Although *S. imbricata* has fungicidal properties, there is little scientific evidence supporting its fungitoxic nature. The present study aimed to analyze the phytochemical composition of the methanolic extract of *S. imbricata* picked from Matrouh, Egypt. Additionally, we examine its antioxidant and antibacterial properties against specific phytopathogenic bacteria and fungi. The findings indicate that the extract yield of *S. imbricata* was 8.37 g/100 g dry matter. It was found that macerating the aerial parts of three species of *Salsola* led to methanolic extracts with yields of 8.51% for *S. oppositifolia*, 7.61% for *S. soda*, and 10.8% for *S. tragus* [38]. Furthermore, Ajaib et al. [39] demonstrated *S. imbricata* fruit produced an aqueous extract with an extraction yield of 11.2%. The methanol bark extract of *S. imbricata* Forssk. revealed a minimum yield of 5.12%. In their study, Osman et al. [40] obtained 112 g of powdered extract from 1,000 air-dried leaves of *S. imbricata*. The extraction process involved refluxing the leaves three times in methanol (75%).

Ajaib et al. [39] conducted a qualitative phytochemical investigation of *S. imbricata* and detected several bioactive components in different extracts. These chemicals include alkaloids, saponins, flavonoids, tannins, reducing sugar anthraquinone, and cardiac glycosides. Saponins, which are a crucial category of secondary metabolites found in plants, carry out several biological functions. Extensive research investigated the antibacterial features of the saponins [41,42]. Research indicates that pathogenic fungi harm chlorophyll-containing cells through the secretion of enzymes that degrade saponins. The ongoing analysis has discovered a substantial amount of saponin in the methanolic extract of *S. imbricata*. Soetan [43] suggests that saponins have a restricted capacity to traverse bacterial cell membranes. Ojinnaka and Kenne [44] documented the extraction of methyl ursolate from the stem of *Myrianthus arboreus*. It is crucial to acknowledge the intricate nature of plant extracts and that the effects may not be only attributed to one component but rather to a group of molecules (with either substantial or modest effects) that interact in an additive or synergistic way [45]. Researchers have discovered that the secondary metabolites of the plant, specifically the total phenolic, flavonoid, and tannin contents, play a crucial role in its capacity to combat free radicals. Hamdi et al. [46] discovered the total phenolic content exhibited significant variation in *Salsola baryosma* herbal materials, ranging from 0.75 to 2.60 mg GAE/g dry weight. Furthermore, the flavonoid content ranged from 0.46 to 0.19 mg/g rutin equivalent to the crude extract. We found that the TPC and TFC were higher than those reported by Shehab and Abu-Gharbieh [47]. The discrepancy in our research findings can be attributed to the variation in plant material used for extraction. In our study, we specifically used the aerial part of the plant, whereas Shehab and Abu-Gharbieh [47] extracted the whole plant, which was of herbal origin and obtained from the desert of Dubai, UAE. Additionally, the *S. imbricata* used in our study was sourced from the desert of El-Hamam located in the northern region of Egypt. The geographic location and plant parts can influence the phytochemical composition of plant extracts. Boulaaba et al. [48] discovered variations in the levels of total phenolic, total flavonoid, and condensed tannin contents in *Salsola kali* leaves, stems, and roots during different stages of development. For instance, the total phenolic content in the leaves reached a maximum of 11.9 mg GAE/g dry weight, while in the stems it was 5.6 mg GAE/g dry weight, and in the roots, it was only 0.79 mg GAE/g dry weight. In another study, *Psychotria peduncularis* exhibited the highest TPC and TFC, measuring 5.57  ±  0.22 mg GAE/g and 1.38  ±  0.06 mg QE/g, respectively [11]. The effectiveness of these phenolic compounds in combating fungus is determined by their composition and concentration. Phenolic chemicals possess antifungal properties and can exert their effects through several mechanisms. They can stop the cell cycle, lower the production of certain gene sequences, speed up the growth of mycelium, change normal metabolic pathways, or kill cells by throwing off their natural redox balance [16,49,50]. To assess the antioxidant activity of the studied halophyte plant, we utilized both phosphomolybdenum and DPPH radical scavenging assays. The antioxidant activity was classified as follows: very strong (IC_50_ < 50 µg/mL), strong (50 ≤ IC_50_ < 100 µg/mL), moderate (100 ≤ IC_50_ < 150 µg/mL), and low (IC_50_ > 150 µg/mL) [51]. According to these criteria, our plant extract exhibited very strong antioxidant activity across both assays, although researchers advise against concluding based on one assay [52].

The observed antioxidant activity in these medicinal plants can be attributed to the presence of phenolic compounds, such as phenolic acids and flavonoids, which exert their effects through the hydrogen-donating properties of their hydroxyl groups [53]. Additionally, these phenolic compounds can chelate metal ions that participate in the production of reactive oxygen species [54]. Our findings are consistent with those reported by Ngbolua et al. [55], who documented the antioxidant activity of *A. manniana*, as well as the results of Tsafack et al. [56], who highlighted the antioxidant properties of *T. mauritianum*. Additionally, our study recorded promising antioxidant activity, compared with the methanolic extracts of medlar leaves exhibiting notable antioxidant activity (69.43%) in the DPPH radical assay and demonstrating antibacterial properties against both Gram-positive and Gram-negative bacteria, particularly showing substantial inhibition against *Staphylococcus aureus* (30.83 mm) [57].

Flavonoids are a diverse group of natural compounds that have shown a wide range of chemical and biological properties. They can remove harmful radicals, reduce allergies, fight viral infections, and reduce inflammation [58]. There is a strong link between the amount of phenolic compounds and the antioxidant capacity. This makes sense since phenolic compounds are known to get rid of free radicals [59,60]. Phenolics can get rid of free radicals if they can donate electrons or hydrogen atoms. This depends on how many hydroxyl groups they have and how they are structured and arranged [61]. Findings about phenolic and flavonoid contents are very important for understanding how antibacterial plant-based medicines work [62,63,64]. Therefore, in the current study, HPLC was used to determine how the amounts of phenolics and flavonoids in plant extracts used for animal feed affect their ability to inhibit fungi. Using HPLC to look at the phenolic and flavonoid contents of the methanolic extract of *S. imbricata* shows a varied phenolic profile with 15 identified compounds. Shehab and Abu-Gharbieh [47] employ HPLC to evaluate the levels of phenolic and flavonoid compounds in the extract of the *S. imbricata* plant. Thirteen components were detected at a wavelength of 280 nm, accounting for 13.904% of the overall makeup. Out of these, there were a total of nine phenolic acids, accounting for 9.734% of the compounds. As part of this, there were coumaric acids (4.251% of the total), two flavonoids (catechin and chrysin), diphenol catechol, and one nonphenolic benzoic acid (2.306% of the compounds). At a wavelength of 330 nm, a total of 8 components were detected, with 7 of them being of flavonoidal origin. Quercetin accounted for 12.692% of the components, whereas rosmarinic acid made up 2.734%. As well high concentrations of phenolic compounds, flavonoids, and carotenoids were found in methanolic extracts from the leaves of medlar [57]. The majority of phytochemicals are classified as phenolic, which refers to substances containing one or more aromatic rings that contain hydroxyl groups. They function as a defensive mechanism against microbial diseases [65]. Moreover, Nayak et al. [66] established that these compounds have a significant role in both reproduction and growth.

A GC–MS analysis of *S. imbricata* extract showed a wide range of compounds. Some of the main ones found were palmitoleic acid, oleic acid, palmitic acid, linoleic acid, stearic acid, methyl palmitate, linoleic acid methyl ester, stigmasterol, β-sitosterol, and phytol. These compounds, known for their varied biological activities, contribute to the potential health benefits of the extract. The presence of fatty acids such as palmitic, palmitoleic, linoleic, and oleic acids suggests the extract’s potential antioxidant and antimicrobial characteristics, supported by existing literature on the positive effects of these fatty acids on cellular health [67,68]. Additionally, the discovery of stigmasterol and β-sitosterol fits with earlier research that showed their antioxidant and anti-inflammatory properties. This supports the idea that the *S. imbricata* extract might be useful for treating health problems [69,70]. Griebel and Zeier [71] revealed that the interaction between *Arabidopsis thaliana* and *Pseudomonas syringae* results in the accumulation of stigmasterol, a phytosterol. This accumulation is a noteworthy metabolic activity that takes place in plants following bacterial leaf infection. Both avirulent and virulent *P. syringae* exhibit increased resistance to stigmasterol buildup. Furthermore, the presence of phytol in the extract adds to its potential antimicrobial and antioxidant capabilities. Phytol has demonstrated antimicrobial activity against various pathogens and antioxidant effects, as evidenced in studies investigating its role in medicinal plants [72,73]. Even though each of the identified compounds has bioactive properties of its own, the way they work together in the complex mixture of the extract may make the biological activities even stronger. In the same manner, Rasheed et al. [74] conducted GC–MS profiling of non-polar *n*-hexane extracts from five *Salsola* species, identifying various compounds. In *S. arabica*, oleic acid predominated, constituting 75.6% of the extract. The *n*-hexane extract of *S. cyclophylla* contained 25 different compounds. The most important ones were 1-octadecene (14.5%), 1-hexadecanol (13.7%), benzoic acid pentadecyl ester (11.3%), and 2,4-di-tert-butylphenol (10.4%). Cinnamaldehyde, -hexyl-, dominated *S. imbricata* extract, comprising 57.2% of the extract, with a total of 13 compounds identified. There were 18 chemicals in *S. inscanescens*, with octacosyl heptafluorobutyrate making up 25.4% of them. The most common fatty acid methyl esters in *S. villosa* were methyl palmitate (26.2%), methyl linoleate (17.8%), and methyl oleate (16.3%). Additionally, phytol was found in the *n*-hexane extracts of *S. villosa* and *S. imbricata*. Fatty acids such as linolenic, oleic, arachidonic, palmitic, and stearic acids were found in the aerial portions of *S. kali*. They also found sterols like β-sitosterol, β-sitosterol-3-*O*-glucoside, sitostanol, stigmasterol, and avenasterol in the upper parts of *S. tetrandra*, *S. rigida*, and *S. longifolia* [75,76]. For instance, a study on *Lantana camara* evaluated its antioxidant and anti-tumor properties, identifying four key compounds, including lantadene A and B. These compounds demonstrated significant antioxidant activity and reduced MCF-7 breast cancer cell viability, with lantadene B exhibiting the most potent anti-cancer effects and inducing cell cycle arrest in the G1 phase [77]. This highlights the potential of exploring plant-derived compounds, like those from *S. imbricata*, for therapeutic applications.

Overall, while our investigation sheds light on the chemical composition and biological activity of *S. imbricata*, we recognize some limitations that result from focusing solely on one species. One significant restriction is that the bioactive compounds and antibacterial activity discovered in *S. imbricata* may not fully represent the phytochemical diversity of other halophytic plants from the Arabian region. Some species, like *Salsola kali*, *Haloxylon salicornicum*, and *Suaeda*, may have new or similar chemicals with stronger bioactive effects. Additionally, environmental factors such as soil composition, climate, and location can substantially impact the phytochemistry of halophytic plants. Future studies should compare *S. imbricata*’s phytochemical profiles and bioactivities to those of other Arabian Peninsula species. This method of comparison could help us find new bioactive chemicals and find out if the biological activities reported are unique to *S. imbricata* or shared by related species. This would help us learn more about the different biological potential of plants that have adapted to living in deserts.

## 5 Conclusion

In conclusion, the methanolic extract from *S. imbricata* aerial parts exhibits promising antioxidant and antimicrobial properties, attributed to its rich phytochemical composition. The extract contains various secondary metabolites, including phenolic acids, fatty acids, and steroids, which contribute to its bioactivity. Antimicrobial tests revealed significant inhibition against several bacterial and fungal strains, with varying responses based on concentration. Further research is warranted to explore the extract’s potential as a natural alternative for agricultural applications.
